# Structure and Dynamics of the Superprotonic Conductor Caesium Hydrogen Sulfate, CsHSO_4_

**DOI:** 10.3390/molecules25061271

**Published:** 2020-03-11

**Authors:** Stewart F. Parker, Hamish Cavaye, Samantha K. Callear

**Affiliations:** ISIS Facility, STFC Rutherford Appleton Laboratory, Chilton, Didcot, Oxon OX11 0QX, UK

**Keywords:** caesium hydrogen sulfate, inelastic neutron scattering, neutron diffraction, Raman spectroscopy, density functional theory

## Abstract

We have investigated caesium hydrogen sulfate, CsHSO_4_, in all three of its ambient pressure phases by total scattering neutron diffraction, inelastic neutron scattering (INS) and Raman spectroscopies and periodic density functional theory calculations. Above 140 °C, CsHSO_4_ undergoes a phase transition to a superprotonic conductor that has potential application in intermediate temperature fuel cells. Total scattering neutron diffraction data clearly show that all the existing structures of this phase are unable to describe the local structure, because they have either partial occupancies of the atoms and/or non-physical O–H distances. Knowledge of the local structure is crucial because it is this that determines the conduction mechanism. Starting from one of the previous models, we have generated a new structure that has no partial occupancies and reasonable O–H distances. After geometry optimisation, the calculated radial distribution function is in reasonable agreement with the experimental data, as are the calculated and observed INS and Raman spectra. This work is particularly notable in that we have measured INS spectra in the O–H stretch region above room temperature, which is extremely rare. The INS spectra have the enormous advantage that the electrical anharmonicity that complicates the infrared spectra is absent and the stretch modes are plainly seen.

## 1. Introduction

Caesium hydrogen sulfate, CsHSO_4_, is a solid acid proton conductor, which operates in the temperature range from 140 to 200 °C. The material has potential application in intermediate temperature fuel cells as a solid electrolyte [1,2].

At ambient pressure, CsHSO_4_ exhibits a complex phase diagram [3], in addition there are several high pressure phases. It is obtained by evaporation of a solution of sulfuric acid and Cs_2_CO_3_. This produces the metastable phase III which transforms to phase II at around 60 °C. On cooling, phase II returns to room temperature without making a transition to phase III. On heating, phase II reversibly transforms to phase I at 141 °C and the material melts at 211 °C. For CsDSO_4_, phase III is not formed and the material crystallises directly into phase II. It reversibly transforms to phase I at 139 °C. All three phases have been studied crystallographically [4,5,6,7,8,9,10,11]; however, the detailed structure of the proton conducting phase and the high pressure phases are still controversial [7,12]. As proton conduction is a localised process, it moves from one site to an adjacent one, it follows that it is crucial to understand the local structure of the material in order to understand the proton conduction. Total scattering neutron diffraction [13] has the twin advantages that it probes both the local structure and is sensitive to hydrogen, as demonstrated by the location of the bridging hydrides in Stryker’s reagent, [HCu{P(C_6_H_5_)}_3_]_6_ [14] and of the surface hydrides on Raney nickel [15]. A study of the local structure of the CsDSO_4_ in phase I, combined with reverse Monte Carlo (RMC) modelling [16], showed that the “deuterons are rather free to move through the cell”.

Phase I shows a high proton conductivity, the superprotonic phase. A two-step mechanism consisting of a reorientation motion of the [HSO_4_]^−^ ion and a proton transfer between the ions along the hydrogen bond axis has been suggested for its proton conduction [16,17]. An alternative mechanism proposes [18] that CsHSO_4_ above 141 °C is not a superprotonic conducting phase but rather that the conduction results from water formed by partial thermal decomposition of CsHSO_4_ to Cs_2_S_2_O_7_:

2CsHSO_4_ → Cs_2_S_2_O_7_ + H_2_O (1)

The vibrational spectroscopy of CsHSO_4_ has been studied by infrared, Raman [12,19,20,21,22,23,24,25] and inelastic neutron scattering [26,27,28] (INS) spectroscopies as a function of both pressure and temperature. Clear differences between the phases are readily apparent using infrared and Raman spectroscopies. Unfortunately, the most diagnostic mode, the O–H stretch, is masked by the effects of strong electrical anharmonicity in the infrared spectrum and it is intrinsically very weak (and usually unobservable) in the Raman.

In INS spectroscopy [29], there are no selection rules, electrical anharmonicity is irrelevant and there is a propensity to observe modes involving proton motion; hence, it may be expected that the O–H stretch should be readily observable by INS spectroscopy. To date, this is not the case. The reason for this lies in the type of spectrometer used and the physics of INS spectroscopy. Since neutrons have mass, an inelastic scattering event results in a significant transfer of both energy (*ω*, cm^−1^) and momentum (*Q*, Å^−1^). The observed intensity, *S*, of an INS transition depends on both *ω* and *Q* and is given by [29]:

(2) $$S(Q,n\omega_{i})\propto \frac{{\left(QU_{i}\right)}^{2n}}{n!}\exp\left(-{\left(QU_{\mathrm{Tot}}\right)}^{2}\right)\;\sigma$$

where *ω_i_* is the *i*th mode at transition energy *ω*, *n* = 1 for a fundamental, 2 for a first overtone or binary combination, 3 for a second overtone or ternary combination etc., *U_i_* is the root mean square displacement of the atoms in the mode. *σ* is the inelastic scattering cross section of the atom and this is both element- and isotope-specific and is ~20 times larger for ^1^H than any other nucleus (including ^2^H). Hydrogen is the lightest element; consequently, its amplitude of vibration (*U_i_*) is the largest of any element and in combination with the exceptionally large incoherent cross section has as a result that scattering from hydrogenous materials is dominated by modes that involve motion of hydrogen. The exponential term in Equation (2) is a Debye-Waller factor, *U*_Tot_ is the total root mean square displacement of all the atoms in all the modes (both internal and external), and its magnitude is in part determined by thermal motion. The type of INS spectrometers (indirect geometry) used to date for the study of CsHSO_4_ cannot provide useful information in the region >2500 cm^−1^ because they have a fixed relationship between momentum transfer and energy transfer such that *Q*^2^ ≈ *ω*/16. Thus, high energy means large momentum transfer (*Q* ~15 Å^−1^ at 3000 cm^−1^) and from (2), the O–H stretch intensity is severely damped by the large Debye-Waller factor. Furthermore, as the intensity of combination modes between the internal and external modes (phonon wings) depend on *Q*^4^, *Q*^6^… their intensity will be much larger than that of the fundamental; the consequences are that the O–H stretch has not yet been observed by INS spectroscopy.

We have shown [30,31] that a different type of INS spectrometer (direct geometry) has considerable advantages for molecular spectroscopy in the region >2000 cm^−1^. In particular, we have shown for a related system, KH_2_AsO_4_ [32], that a detailed understanding of the dynamics is possible from INS spectroscopy. The ability of direct geometry spectrometers to access low momentum transfer at large energy transfer means that the O–H stretch region is readily accessible, because the Debye-Waller factor is greatly reduced and the intensity of the overtones and combinations (*n* > 1) can be minimised. The access to small *Q* also means that the detrimental effect of temperature may be sufficiently reduced as to allow measurements at or above room temperature, as was demonstrated for a reacting system [33].

The aim of this paper was to exploit a synergistic combination of vibrational spectroscopy, total scattering neutron diffraction and ab initio studies to provide new insight into the structure and dynamics of CsHSO_4_. The structure of the paper is that we first investigate the vibrational spectroscopy of CsHSO_4_ in all three phases and then consider the structure of phase I. We compare the results of periodic density functional theory calculations with our experimental data for phases II and III to show that the calculations are reliable. This gives confidence that the same methods applied to phase I are valid.

## 2. Results and Discussion

### 2.1. CsHSO_4_ Spectroscopy

**Figure 1:**
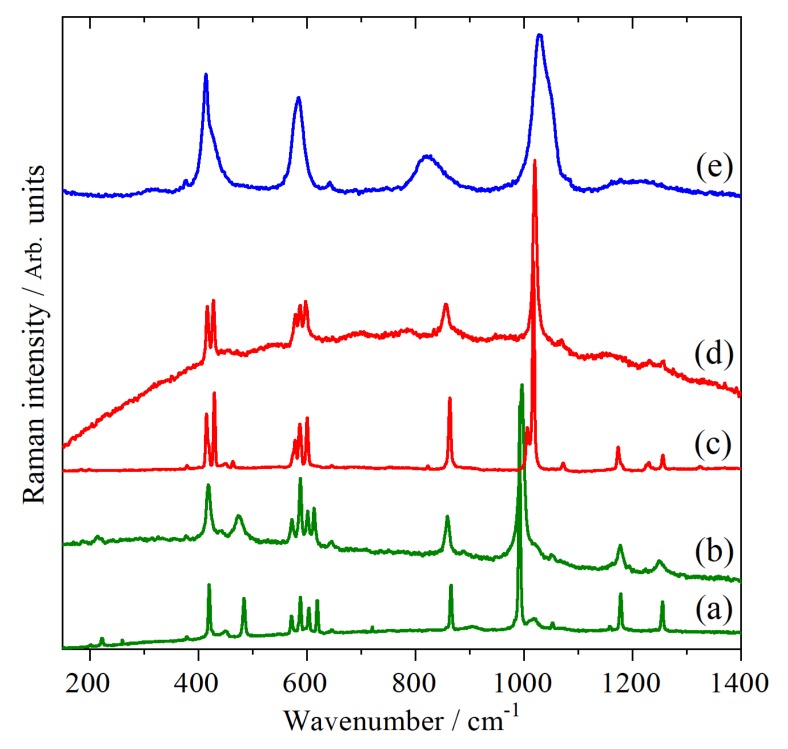
Raman spectra of CsHSO_4_ in: phase III at (**a**) 10 K and (**b**) at 290 K, phase II at (**c**) 10 K and (**d**) at 300 K and phase I (**e**) at 430 K.

The Raman spectroscopy of CsHSO_4_ has been extensively studied in all three phases [19,20,21,22,23,24,25]. Our high temperature results, >300 K, are completely in agreement with the literature and typical spectra are shown in Figure 1. Low temperature data (10 K) for phases II and III have not been reported previously; it can be seen that apart from a significant sharpening of all the bands and some minor shifts of a few wavenumbers, there is no difference between the high and low temperature data. This is consistent with there being no phase changes below room temperature. It is noticeable that the spectra are markedly different in each phase, in particular, the sulfate symmetric stretch at ~1000 cm^−1^ undergoes a progressive shift to higher wavenumber: 996 cm^−1^ (III at 290 K) → 1019 cm^−1^ (II at 300 K) → 1028 cm^−1^ (I at 430 K); the width also approximately doubles in phase I. These changes provide an unambiguous method to identify the phase. With our capability to carry-out simultaneous Raman and neutron scattering measurements [34], this provides an independent check that we are observing the correct phase.

**Figure 2:**
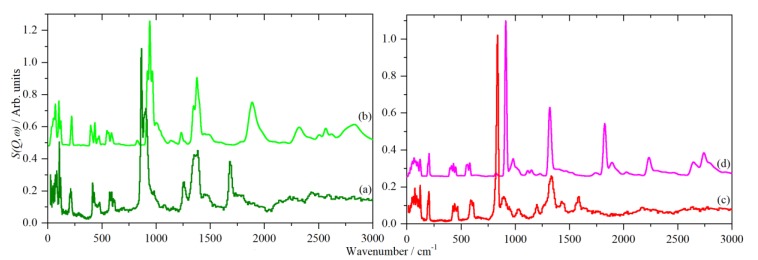
INS spectra of CsHSO_4_ at 10 K. (**a**) Observed and (**b**) calculated for phase III. (**c**) Observed [27] and (**d**) calculated for phase II.

Figure 2 shows the INS spectra of phases III and II recorded on TOSCA [35] at 10 K, together with the spectra generated from calculations of the complete Brillouin zone based on the structures of Itoh et al. [6] (phase III) and Belushkin et al. [8] (phase II). For both phases, all real modes are found across the entire Brillouin zone, consistent with mechanically stable materials.

Phase III has been the subject of a comprehensive spectroscopic and computational investigation by Krzystyniak et al. [28]. Our INS spectrum is of higher quality as it was recorded with a later generation of the instrument, otherwise both the observed and calculated (for the same choice of functional) spectra agree with the previous work [28]. For phase II, there are only empirical assignments available [27].

For both spectra, the assignments are similar: translational modes below 200 cm^−1^, sulfate bending modes in the range 200–700 cm^−1^, out-of-plane S–O–H bend at ~800 cm^−1^ and in-plane S–O–H bend at ~1320 cm^−1^. The modes at >1500 cm^−1^ are overtones and combinations of the bending modes. Table 1 lists the observed and calculated (at the Brillouin zone Γ-point) transition energies with assignments.

The calculated spectra are in reasonable agreement with the experimental data, the only serious discrepancy is the out-of-plane S–O–H bend which are calculated to be too high by ~80 cm^−1^ in both cases. This also means that any overtones or combinations that include this mode will also be too high in wavenumber. A mismatch between observed and calculated transition energies for modes that involve hydrogen bonding is not uncommon, e.g., H_2_O_2_ [36] and aromatic amines and their hydrochlorides [37].

**Figure 3:**
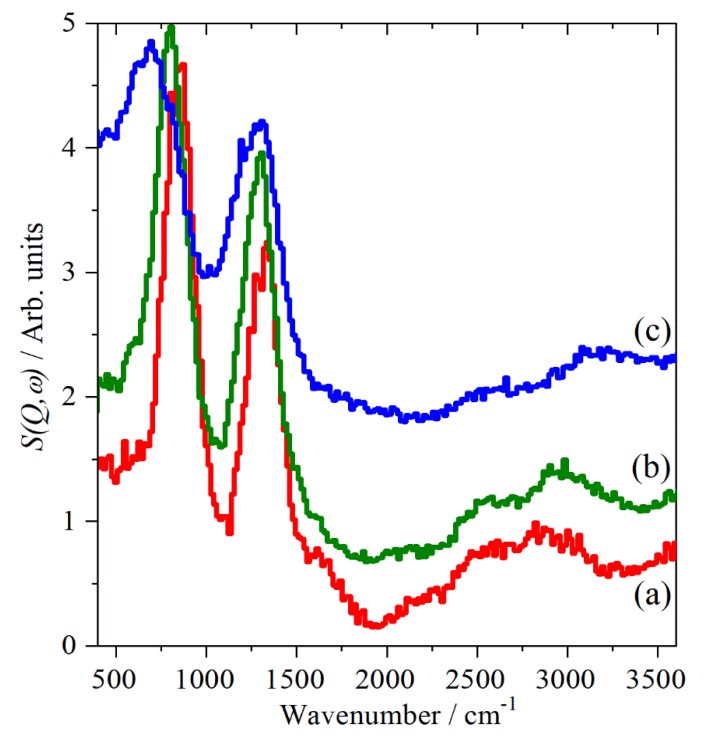
INS spectra recorded with MAPS of CsHSO_4_ with 4840 cm^−1^ incident energy. (**a**) Phase III at 300 K, (**b**) phase II at 300 K and (**c**) phase I at 430 K. The spectra are offset for clarity.

Figure 3 shows the INS spectra recorded on MAPS [30] at or above room temperature. This enables us to access the O–H stretch region and is the first time that this has been clearly observed without the complications of electrical anharmonicity [21]. It is apparent that there are two peaks: one at ~2580 cm^−1^ that is almost phase-invariant and a second that progressively shifts to higher energy: 2870 cm^−1^ (III at 290 K) → 2950 cm^−1^ (II at 300 K) → 3185 cm^−1^ (I at 430 K). In the lower energy region, the two strongest peaks are assigned to the in-plane (~1320 cm^−1^) and out-of-plane (~800 cm^−1^) S–O–H bend. The former is largely phase-invariant, the latter shows a progressive shift to lower energy. The shifts in the O–H stretch and the out-of-plane S–O–H bend are consistent with a weakening of the hydrogen bonding in the sequence III → II → I.

A key observation from the spectra of phase I is that there is no evidence for water being generated via Equation (1) (or any other process). While it would be possible to assign the O–H stretch modes to water, although the transition energies are much lower than usual, the absence of the H–O–H scissors mode at ~1600 cm^−1^ eliminates the possibility that water is present and discounts this model [18] for the proton conduction.

For both phases III and II there are four formula units in the primitive cell [6,8], hence, there are four O–H stretch modes. In both phases, there are two hydrogen bonded chains, each with two OH groups. The mode animations show that the lower energy mode is where both OH move in-phase in the same chain and the higher energy one where they both move out-of-phase in the same chain.

However, the transition energies are calculated as: 2499, 2501, 2619, 2651 cm^−1^ (phase III) and 2680, 2684, 2797, 2831 cm^−1^ (phase II). This suggests two possible assignments: the two modes are the in- and out-of-phase components of the O–H stretch or the lower energy mode is a combination or overtone and the higher energy mode is an unresolved quartet of the O–H stretches. We note that the calculated splitting between the in- and out-of-phase components is ~100 cm^−1^; however, the experimental difference between the two observed features is ~300 cm^−1^.

**Figure 4:**
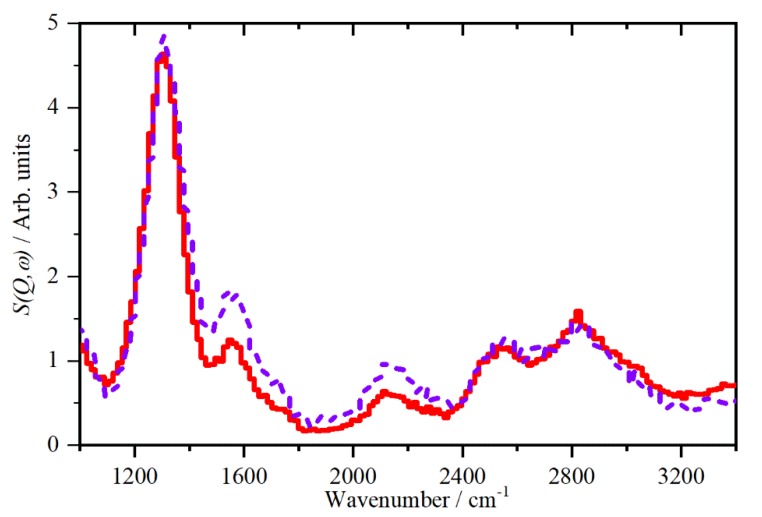
INS spectra recorded with MAPS of CsHSO_4_ in phase II at 10 K. The solid line is for the range 4 ≤ *Q* ≤ 10 Å^−1^ and the dashed line for 10 ≤ *Q* ≤ 16 Å^−1^.

As previously seen for KH_2_AsO_4_ [32] and proton conducting oxides [38] by examining the *Q*-dependence of the modes, fundamentals can be distinguished from overtones and combinations. Figure 4 shows the INS spectrum of CsHSO_4_ in phase II at 10 K recorded on MAPS, which can measure spectra as a function of both *Q* and *ω* [30]. The two traces correspond to low *Q* (4 ≤ *Q* ≤ 10 Å^−1^), which emphasises fundamentals (*n* = 1, Equation (2)), and high *Q* (10 ≤ *Q* ≤ 16 Å^−1^), which emphasises higher order transitions (*n* ≥ 2, Equation (2)). It is apparent that the modes at ~1600 and 2100 cm^−1^ are enhanced in the high *Q* data, thus, they must be *n* ≥ 2 transitions and are readily assigned as the first overtone of the out-of-plane S–O–H bend and the combination of the in- and out-of-plane S–O–H bend, respectively. In contrast, the bands at 2550 and 2830 cm^−1^ are largely unchanged, although there is a small increase in the 2550 cm^−1^ mode. This mode occurs at almost twice that of the in-plane bend, and supported by the poor agreement with the calculations, we assign this as the first overtone of the in-plane bend and the higher energy mode as the four unresolved O–H stretch fundamentals. The *Q*-dependence is not as marked because the *Q*-range is much reduced at this energy transfer.

### 2.2. CsDSO_4_ Spectroscopy

**Figure 5:**
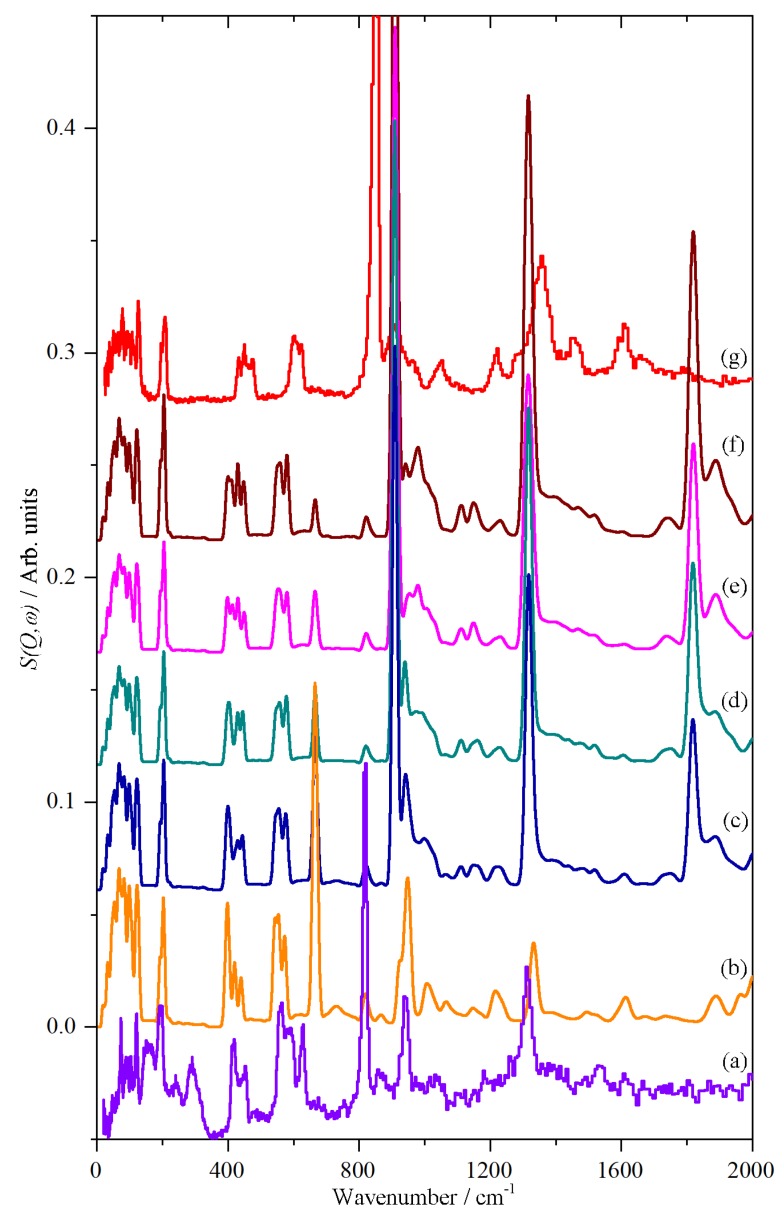
Observed and calculated INS spectra of CsHSO_4_ isotopomers in phase II. (**a**) “CsDSO_4_” experimental spectrum, (**b**) CsDSO_4_ calculated, (**c**) CsH_0.25_D_0.75_SO_4_ calculated, (**d**) CsH_0.5_D_0.5_SO_4_ (D in different chains) calculated, (**e**) CsH_0.5_D_0.5_SO_4_ (D in the same chain) calculated, (**f**) CsH_0.75_D_0.25_SO_4_ calculated and (**g**) CsHSO_4_ experimental spectrum [27]. The ordinate scale relates to (**b**–**f**) and are in the ratios: 1(**b**):0.55(**c**):0.33(**d**–**f**).

As noted in the Introduction, for deuterium contents >50% CsDSO_4_ does not form phase III and the material crystallises directly into phase II [7]. Figure 5a shows the measured INS spectrum of “CsDSO_4_” together with the calculated spectra of various isotopomers, Figure 5b–f and that of CsHSO_4_ in phase II, Figure 5g. There are four formula units in the primitive cell of phase II, consisting of two H-bonded chains. We have calculated the spectra of the D4 (Figure 5b), D3H (Figure 5c), D2H2 (Figure 5d,e), H3D (Figure 5f) and H4 (Figure 5g). For the D2H2 species there are two possible isotopomers: one with the two D atoms in the same chain and one where they are in different chains. However, the spectra are very similar. The S–O–D out-of-plane bend is calculated to be at 665 cm^−1^ (observed at 630 cm^−1^) and is seen strongly in the D4 species, but the intensity relative to the adjacent ν_4_ O–S–O bend mode rapidly diminishes with increasing ^1^H content.

It is apparent that there is a significant ^1^H impurity present, as shown by the band at 820 cm^−1^ in the measured spectrum, Figure 5a. Comparison with the calculated spectra, suggests that it is in the range 50–75% D.

### 2.3. CsHSO_4_ Structure

**Figure 6:**
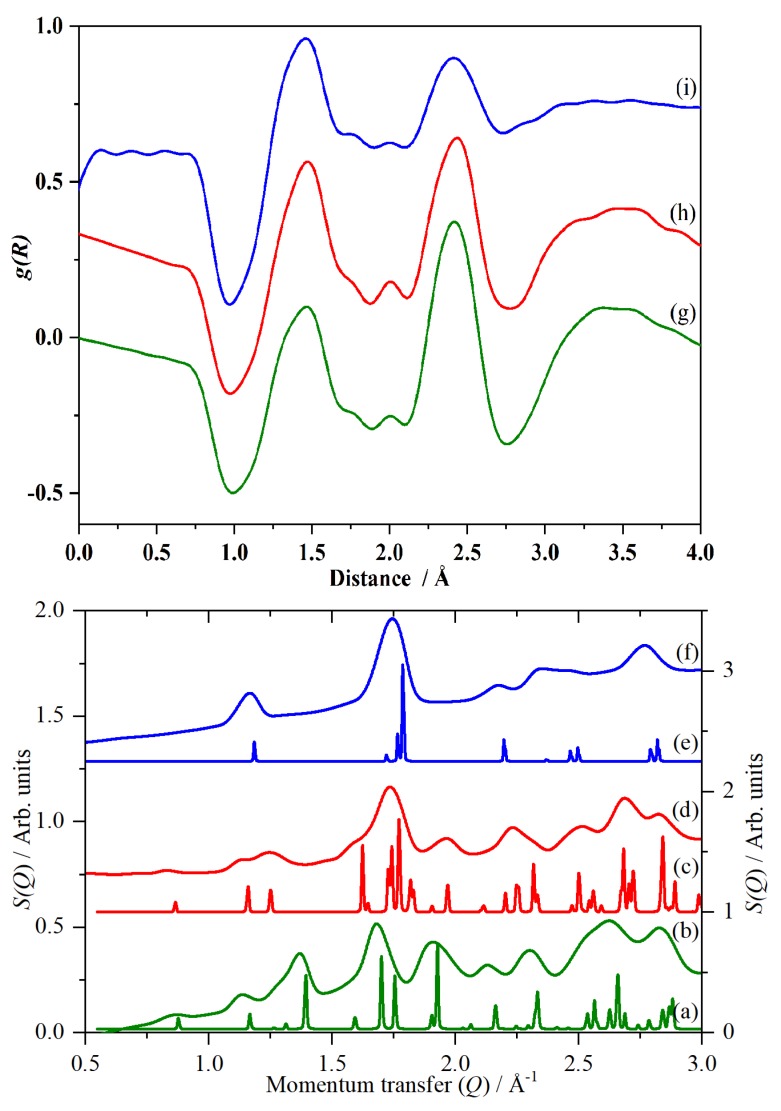
(**a**–**e**): Diffraction patterns of CsHSO_4_ in: phase III at 290 K (**a**) calculated [6] and (**b**) this work. Phase II at 300 K (**c**) calculated [8] and (**d**) this work. Phase I at 430 K (**e**) calculated [9] and (**f**) this work. (**g**–**i**): Radial distribution functions, *g(R)*, of CsHSO_4_: (**g**) phase III, (**h**) phase II and (**i**) phase I. These are generated from (**b**,**d**,**f**), respectively. Note that (**d**,**h**) are 1.3× ordinate expanded. In the lower panel, the right hand side is the ordinate scale for (**a**,**c**,**e**); the left is for (**b**,**d**,**f**).

While the primary purpose of the SANDALS [39] instrument at ISIS [40] is the investigation of local structure, it is actually a low resolution powder diffractometer, with the detector coverage biased to forward scattering. This enables phase identification from the low momentum transfer, *Q*, diffraction data (which corresponds to the average structure) recorded simultaneously with the Raman spectra. As Figure 6 shows, there is good agreement between the data and the literature.

Figure 6g–i show the radial distribution functions, *g(R)*, of CsHSO_4_ in the three phases, generated from the data shown in Figure 6b,d,f. ^1^H has a negative scattering length (it undergoes a 180° phase change on scattering); all the other elements present have positive scattering lengths, thus, the oxygen-to-hydrogen correlation results in a negative-going peak (since −ve × +ve = −ve) and the negative-going peak at 1 Å is the O–H distance. This is the first direct measurement of the O–H distance in phase I.

The two positive peaks at 1.46 Å and 2.43 Å are the S–O and the intramolecular O⋯O distances of the [HSO_4_]^−^ ion respectively and are phase-invariant as would be expected for covalent bonds.

**Figure 7:**
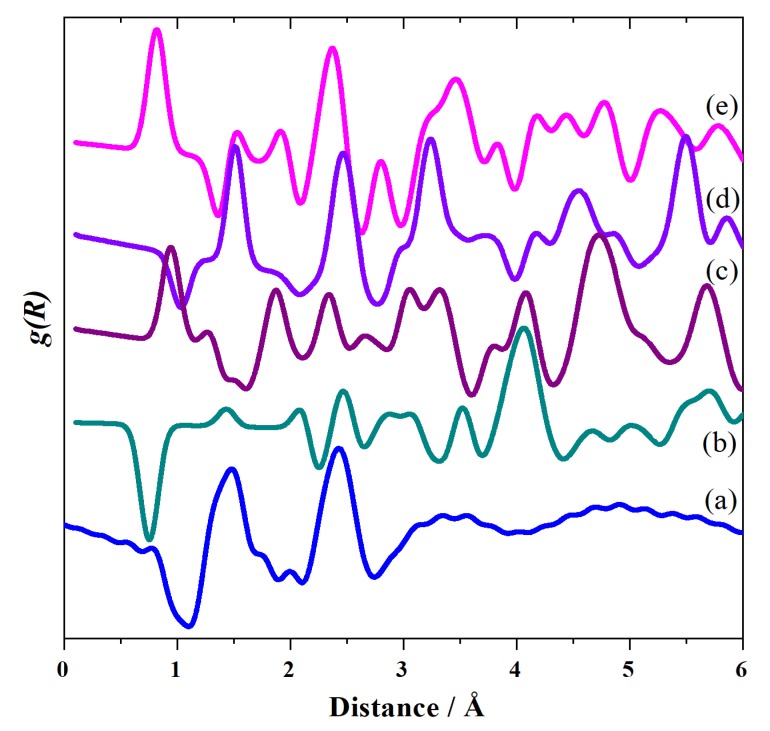
Radial distribution functions, *g(R)*, of CsHSO_4_ in phase I: (**a**) this work and generated from the structures of: (**b**) Merinov [11], (**c**) Nozik et al. [4], (**d**) Beluskin et al. [7] and (**e**) Jirak et al. [9].

The structure of phase I of CsHSO_4_ has been investigated several times and there is agreement that it is tetragonal (space group *I*4_1_/*amd*) [4,7,9,11,16]. However, all of the studies except one (which used CsDSO_4_ [16]) have focussed on the average structure and, as may be seen from Figure 7, this is a poor description of the local structure. Either (or both) there are peaks at non-physical distances (1 Å <) or the agreement with the experimental data is poor. The discrepancies arise because all the structures have oxygen atoms with partial occupancies (so the O⋯O distance is very short) and the hydrogen atoms are in high symmetry positions located midway between two oxygen atoms, resulting in O–H distances of ~1.5 Å.

**Figure 8:**
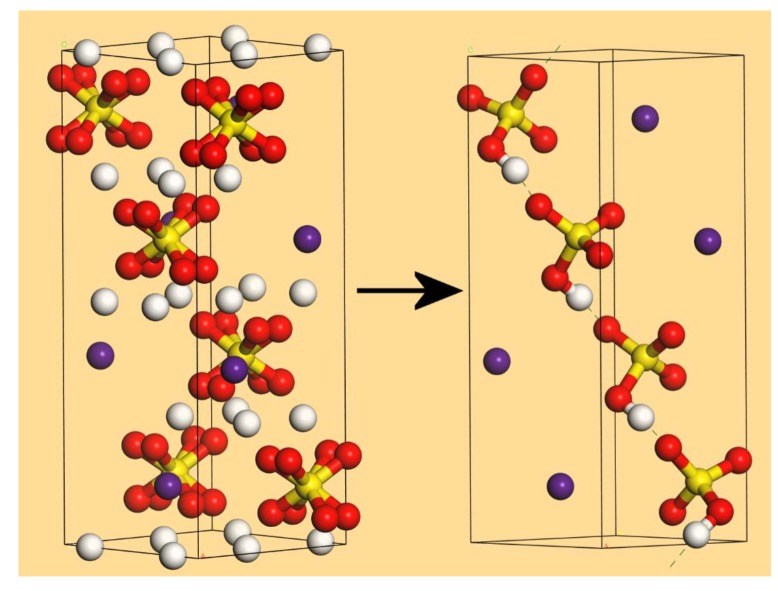
Left: the structure of CsHSO_4_ in phase I by Jirak et al. [9]. Right: the structure after choosing one orientation of the sulfate ion and geometry optimisation. (white = hydrogen, red = oxygen, yellow = sulfur, purple = caesium).

The differences between the various models proposed for phase I are comprehensively discussed by Chisholm and Haile [41]. On the basis of a new measurement of the entropy of the transition to the high-temperature phase of CsHSO_4_, they concluded that the Jirak et al. [9] model was the only one that could account for the entropy change. This structure is shown in the left side of Figure 8. As noted earlier, this structure has partial occupancies for the oxygen and hydrogen atoms. Choosing one oxygen atom at random immediately determines the other three of the sulfate ion; then, selecting the hydrogen atom closest to one of these oxygen atoms and following the modified “ice rules” [41] (only one hydrogen bound to oxygen and only one hydrogen bond per sulfate, which must involve an oxygen that is only bonded to sulfur) leads to structures with chains of hydrogen bonded [HSO_4_]^−^ ions running through the structure. Assigning all of these atoms full occupancy and deleting all the other hydrogen and oxygen atoms (the S and Cs locations are fixed by symmetry) generates a possible structure. One such structure is shown in the right side of Figure 8 after geometry optimisation. This has *P*1 symmetry; the “real” structure will comprise of all such possible arrangements so as to generate the observed tetragonal symmetry.

**Figure 9:**
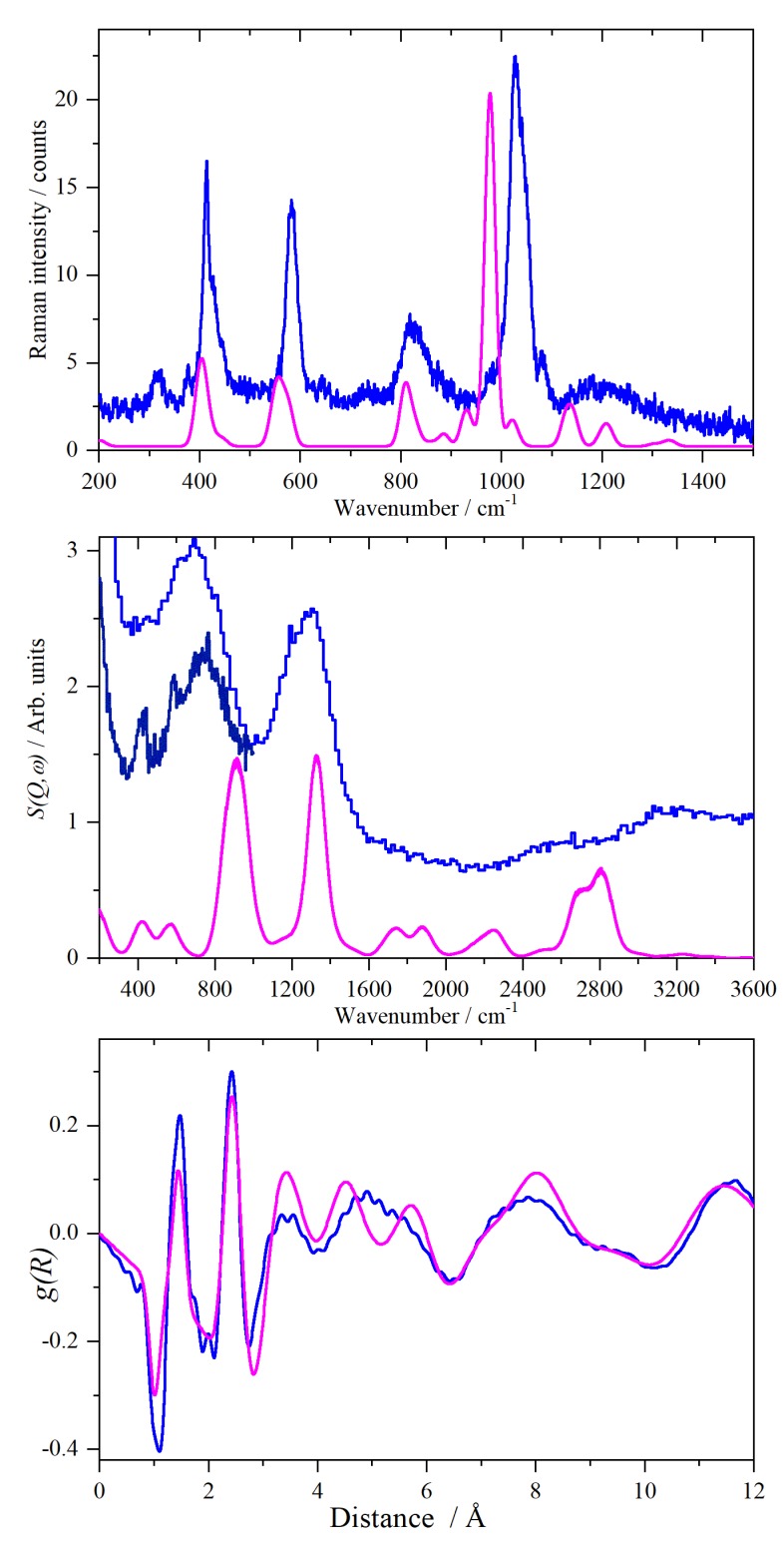
Comparison of experimental (blue) and the calculated (magenta, generated from the structure shown in the right-hand side of Figure 8) data of CsHSO_4_ in phase I at 430 K. Bottom: radial distribution function, *g(R)*. Middle: INS spectrum (experimental MAPS spectra recorded with 650 meV and 150 meV (navy) incident energy). Top: Raman spectra.

Figure 9 compares the experimental data with that generated from the model shown in the right-hand side of Figure 8, for the local structure, INS and Raman spectra, respectively. It can be seen that all three show reasonable agreement between experiment and the model. In particular, the radial distribution function captures the local structure. The calculated INS spectrum shows too much intensity in the O–H stretch region, but this is a consequence of the neglect of the Debye-Waller factor, which is significant at 430 K, in the calculated spectrum. In the Raman spectrum, the intensity of the ν_1_ S–O symmetric stretch (observed at 1027 cm^−1^, calculated at 978 cm^−1^) is overestimated; otherwise, the agreement is acceptable.

## 3. Materials and Methods

CsHSO_4_ was prepared by slowly dropping a stoichiometric quantity of H_2_SO_4_ (95–98% diluted in water) to a stirred solution of Cs_2_CO_3_ (99%). The resulting solution was then evaporated to dryness overnight. CsDSO_4_ was prepared similarly from D_2_SO_4_ (96–98% in D_2_O, 99.5 atom% D) and Cs_2_CO_3_ in D_2_O. All the chemicals were purchased from Sigma-Aldrich (Gillingham, Dorset, UK) and used as received.

INS spectra were recorded using the MAPS [30] and TOSCA [35] spectrometers at ISIS (Chilton, Oxfordshire, UK) [40]. On TOSCA the resolution is ~1.25% of the energy transfer across the entire energy range, while on MAPS, under the conditions used here, it is ~1.5% of the incident energy at the largest energy transfer and degrades with decreasing energy transfer. Thus, TOSCA provides excellent energy resolution at energy transfers < 1200 cm^−1^, at larger energy transfer MAPS provides better resolution by virtue of the access to low *Q*, this also enables studies above room temperature. TOSCA and MAPS are highly complementary and enable the complete range of interest, 0–4000 cm^−1^, to be covered with good resolution.

Time-of-flight neutron diffraction (ND) measurements were performed using the diffractometer, SANDALS [39], at ISIS (Chilton, Oxfordshire, UK) [40] A full set of experimental corrections and an absolute normalisation were made using the standard Gudrun [42] software. The diffractograms were analysed using PDFgui [43].

Raman spectra were recorded simultaneously with the neutron data using the special Raman-enabled centrestick [34] in order to provide unambiguous phase confirmation.

Periodic density functional theory (periodic-DFT) calculations were carried out using a plane wave basis-set and pseudopotentials as implemented in the CASTEP code (version 17.21) [44,45]. The generalised gradient approximation Perdew-Burke-Ernzerhof (PBE) functional was used in conjunction with optimised norm-conserving pseudopotentials with a plane-wave cut-off energy of 830 eV. For all three phases a Monkhorst-Pack grid of 6 × 6 × 4 (36 k-points for phases II and III, 72 for phase I) was used. As a prerequisite to any lattice dynamics calculation a full geometry optimisation of the internal atomic co-ordinates was performed, the calculations were converged to |0.0074| eV Å^−1^. Phonon modes were calculated using the density-functional perturbation-theory [46]. The output of the phonon calculation includes infrared intensities and the atomic displacements of the atoms in the mode. Raman intensities were calculated using a hybrid method combining density functional perturbation theory with finite displacements [47]. Calculations of the isotopic species used the program PHONONS (version 1) [48]. The visualisations of the modes were carried out in Materials Studio 2017 (version R2(17.2.0.1626), BIOVIA) [49] and the INS spectra were generated with ACLIMAX (version 6.0.0 LE) [50] or AbINS (version 1) [51].

## 4. Conclusions

The total scattering neutron diffraction data clearly shows that all the existing structures for phase I of CsHSO_4_ are unable to describe the local structure. Knowledge of this is crucial because it is the local structure that determines the conduction mechanism. Following the conclusion of Chisholm and Haile [41] that only the Jirak et al. [9] model can account for the entropy change, we have tested this by deriving a structure that has no partial occupancies. After geometry optimisation, the calculated radial distribution function is in reasonable agreement with the experimental data, as are the calculated and observed INS and Raman spectra. We recognise that this is only one possible structure and it is conceivable that an ensemble of such structures or a large supercell encompassing multiple possibilities would give better agreement. However, this is beyond our computational resources. The model proposed in the right-hand side of Figure 8 offers a simple path for proton conduction by a Grotthuss-like mechanism of proton jumps.

Our results show no evidence for the generation of water via decomposition of CsSO_4_H to Cs_2_S_2_O_7_, Equation (1) [18].

This work is particularly notable for the spectra shown in Figure 3: INS spectra in the O–H stretch region at realistic temperature (300 K and above) are extremely rare—to our knowledge, this is only the second such example ([33] being the first). They have the enormous advantage that the electrical anharmonicity that complicates the infrared spectra is absent and the modes are plainly seen.

## Figures and Tables

**Table 1:** Observed and calculated ^1^ transition energies (cm^−1^) with assignments for CsHSO_4_ in phases III, II and I.

Phase III			Phase II			Phase I			Assign ^3^
Cal ^1^	INS ^2^	Raman	Cal	INS	Raman	Cal	INS	Raman	
	10 K	10 K		10 K	10 K		430 K	430 K	
0–96	32–88 w		0–96	25–1010 w		0–93			Translations
102	107 m		103	105 w		101			HSO_4_ Libration
116	120 w		123	123 w		117			HSO_4_ Libration
219	202,212,218 w,w,w	221 w	199	192,203 sh,w		194			HSO_4_ Libration
399			398			404		414 s	ν_2_ O–S–O bend
441		421 s	432	425,441,462 m,m,m	415,430 s,s	426	430 m	432 sh	ν_2_ O–S–O bend
547			549			552			ν_4_ O–S–O bend
560	572,589 m,m	570,589 m,s	561	590 m	579,587 m,s	561			ν_4_ O–S–O bend
593	606,614 m,m	605,619 m,m	580	600,612 m,m	601 s	577		582 s	ν_4_ O–S–O bend
822		866 s	821		863 s	818		823 m,br	S–OH stretch
942	865,902 vs,s		913	830 vs		904	746 s		S–O–H oop bend
984		995 vs	1006	1032 w	1016 vs	1011		1027 vs	ν1 S–O sym stretch
1133		1178 s	1146		1173 m	1140		1177 w,br	ν3 S–O asym stretch
1252		1255 m	1215		1256 m	1210		1210 w,br	ν3 S–O asym stretch
1372	1368 s		1320	1332 s		1324	1295 s		S–OH ip bend
2589			2733			2757	3185		O–H stretch

^1^ As calculated by CASTEP. There are four formula units in the primitive cell of all phases. Thus, each mode of the isolated ion gives rise to four components in the solid state spectrum. The value given is the average of the factor group quartet. ^2^ s = strong, m = medium, w = weak, sh = shoulder br = broad, v = very. ν refers to the isolated sulfate ion modes. ^3^ oop = out-of-plane, ip = in-plane, sym = symmetric, asym = asymmetric.
